# Consultations with complementary and alternative medicine practitioners by older Australians: results from a national survey

**DOI:** 10.1186/1472-6882-13-73

**Published:** 2013-04-02

**Authors:** Laurann Yen, Tanisha Jowsey, Ian S McRae

**Affiliations:** 1 Menzies Centre for Health Policy & Australian Primary Health Care Research Institute, Ian Potter House, Australian National University, Acton, ACT, 0200, Australia

**Keywords:** Complementary, Health practitioner, Self-manage, Chronic illness, Older

## Abstract

**Background:**

The use of complementary and alternative medicines (CAM) and CAM practitioners is common, most frequently for the management of musculoskeletal conditions. Knowledge is limited about the use of CAM practitioners by older people, and specifically those with other long term or chronic conditions.

**Methods:**

In 2011 we conducted an Australia wide survey targeting older adults aged over 50 years (n = 2540). Participants were asked to identify their chronic conditions, and from which health professionals they had ‘received advice or treatment from in the last 3 months’, including ‘complementary health practitioners, e.g. naturopath’. Descriptive analyses were undertaken using SPSS and STATA software.

**Results:**

Overall, 8.8% of respondents reported seeing a CAM practitioner in the past three months, 12.1% of women and 3.9% of men; the vast majority also consulting medical practitioners in the same period. Respondents were more likely to report consulting a CAM practitioner if they had musculoskeletal conditions (osteoporosis, arthritis), pain, or depression/anxiety. Respondents with diabetes, hypertension and asthma were least likely to report consulting a CAM practitioner. Those over 80 reported lower use of CAM practitioners than younger respondents. CAM practitioner use in a general older population was not associated with the number of chronic conditions reported, or with the socio-economic level of residence of the respondent.

**Conclusion:**

Substantial numbers of older Australians with chronic conditions seek advice from CAM practitioners, particularly those with pain related conditions, but less often with conditions where there are clear treatment guidelines using conventional medicine, such as with diabetes, hypertension and asthma. Given the policy emphasis on better coordination of care for people with chronic conditions, these findings point to the importance of communication and integration of health services and suggest that the concept of the ‘treating team’ needs a broad interpretation.

## Background

Kiat and colleagues recently observed that the use of complementary & alternative medicines (CAM) can have significant interactions with standard prescription medicines for people living with cardiovascular disease (CVD) and argue the importance of mainstream healthcare practitioners being able to advise consumers about CAM use [1]. They note estimates that as many as 42% of people with CVD take CAM [1]. Many people use CAM to promote health and to manage health conditions, including chronic and long term conditions [2-5], but it is sometimes difficult to distinguish in the literature between the use of CAM, which can be obtained from a variety of sources, including high street pharmacies and supermarkets, from the use of services provided by a range of complementary or alternative health practitioners (CAM practitioner). In addition to the possible interactive effects in combining CAM with prescribed medicines, seeking the advice or treatment of a CAM practitioner such as a naturopath or acupuncturist raises implications for people with chronic conditions in ensuring that care management is both coordinated and effective across the range of practitioners consulted. Limited information is available about the use of CAM practitioners by older people, amongst whom chronic illness is most common.

Based on survey interviews with 3004 people in 1993, MacLennan and colleagues reported in 1996 that one in five people in Australia (20.3%) had ever attended a non-medically trained CAM practitioner [6]. Eighteen years after the study was undertaken the works of MacLennan and colleagues remain highly cited as the authorities on prevalence of CAM and CAM practitioner use in Australia [7-9]. More recently, in 2003 Adams and colleagues reported findings from the Australian Longitudinal Study on Women’s Health, showing that women in the middle aged group of 45–50 years of age were more likely than younger (18–23 years) or older women (70–75 years) to have consulted a CAM practitioner in the last 12 months. In addition, in all age groups, users of CAM were more likely to reside in non-urban areas, have more symptoms and illness, and be higher users of mainstream healthcare practitioners [10,11]. They found that 28% of women in the middle aged group and 15% of women in the older age group reported consulting CAM practitioners [10]. Xue and colleagues [12] note the CAM industry is growing and is now a multi-billion dollar industry. They report that 44.1% of adults in their Australian-wide study reported visiting a CAM practitioner in the past twelve month period, with females more likely than males to use CAM [12]. Robinson & McGrail’s 2004 literature review found several studies that suggest CAM use may be even higher [13].

The majority of studies of CAM use have been carried out in the US, including, for example, work by Goldstein, who has reported, inter alia, on the use of CAM among California adults with, and without cancer [14], and with Hsiao et al. [15] has explored the significance of ethnic background in the use of CAM. In a narrative review relating to CAM use in the general population, Bishop and Lewis [16] conclude that knowledge in the area would be well served by further research exploring specific issues, and suggest looking at predictors of particular CAM use, and the association between CAM use and individual demographic or health characteristics.

Adams et al. (2010) examined the use of CAM and CAM practitioners using participants in a cohort study and found that those using CAM practitioners tended to be younger, female, with higher education levels and above average incomes [17]. This study also reported use of CAM practitioners by condition: respondents with non-arthritic joint pain; and those with mild to moderate depressive symptoms were the more likely to consult a CAM practitioner. In Kristofferson’s Norwegian study of CAM practitioner use, 9.5% of people with no previous diagnosis of cancer or coronary heart disease reported having seen a CAM practitioner in the past 12 months. Almost 8% of people with a previous diagnosis of cancer and 6.4% of people with coronary heart disease reported the same [18]. These numbers are slightly higher than Greenfield and colleagues’ British study of CAM use (including CAM practitioners), where 8.1% of survey respondents reported using CAM to self-manage coronary heart disease [19].

Thomas [20] in the UK found that 71% of CAM practitioner consultations related to musculoskeletal conditions. We were able to identify only one study reporting the use of CAM practitioners according to chronic condition within a general population that specifically included older people [21]. In that study, Al-Windi [21] reported that being middle-aged (25–64 years) and having a diagnosed chronic condition were significantly associated with higher use of CAM practitioners. In a study of provider based CAM use, Sirois[22] found that those factors predicting CAM provider use in the general population; being female, having a higher education level and a higher number of comorbid conditions; held across three illness groups they included, arthritis, inflammatory bowel disease and mixed chronic conditions.

In this paper, we ask what factors are associated with use of CAM practitioners by older Australians living in the community, and discuss those findings in the context of chronic illness management.

## Methods

In February 2011 the Serious and Continuing Illness Policy and Practice Study (SCIPPS) conducted an Australia wide mail survey targeting people aged over 50 years, about time use and co-ordination in relation to chronic illness. The survey design, the sampling method, the survey process and analytic method are outlined below.

### Survey design

The survey was designed to cover three main areas. The first elicited information on standard demographic variables. The second related to the respondent’s health, and asked respondents for their perceptions of their own health using the standard single question measure of self-assessed health. In addition, respondents were asked to nominate chronic diseases with which they had been formally diagnosed, including those from a list of common chronic conditions. Respondents were asked about their health services use, including type and number of health professionals consulted in the previous three months. The final section contained a series of questions to obtain information about co-ordination of care and time spent on health related activity.

The questionnaire was piloted with 18 members of a local health service consumer network, who suggested changes to terminology, simplification of questions and shortening the survey. The amended survey was completed by a further 28 people who were respondents to a previous survey of older Australians by the same team and had indicated their willingness to be involved in further research studies. No further changes were suggested.

### Sample

People aged over 50 years with one or more of Type 2 Diabetes, Chronic Obstructive Pulmonary Disease and Cardiovascular disease were the subject group of a wider study of which this work is part, the Serious and Continuing Illness Policy and Practice Study. To ensure that we were able to obtain a good understanding of chronic illness experience, this participant sample was drawn from members aged over 50 years of three organisations: one which broadly represents all older Australians and two which relate to particular disease conditions.

The three sample pools were: National Seniors Australia, (a non- profit organisation representing Australians aged 50 and over with 285,000 members), the National Diabetes Services Scheme (NDSS) (a government funded service delivering subsidised diabetes supplies, information and support for people with diabetes with 280,000 registrants aged over 50) and the Australian Lung Foundation (a non-profit, advocacy, education, fund raising and support organization with over 14,000 members). We refer to respondents as being part of either the ‘NSA sample’, the ‘Diabetes sample’ or the ‘Lung sample’.

A sample of 5,000 members was drawn from the NSA members, stratified by State of residence and age with an oversampling of older members to increase the proportions with chronic disease. A sample of 2,500 registrants aged 50 years or over was drawn from the NDSS register, stratified by State, age and gender with no oversampling as the scheme operates specifically to subsidise costs for persons with diabetes. A sample of 3,062 members of the Australian Lung Foundation was also drawn, comprising all their members with chronic obstructive pulmonary disease (COPD).

### Survey process

The survey was mailed out to the selected sample, with information about the survey in a letter accompanying the survey form. This letter informed recipients about the reason for the survey and what it hoped to achieve, that the survey was voluntary, and provided contact information to enable the recipient to contact the research leader or the relevant Ethics committee with any concerns. Recipients were offered a choice of responding on line, using Survey Monkey®, a proprietary survey tool, or by completing the survey form and returning it by prepaid post. Recipients were informed that return of the survey was taken to signify consent to take part.

Study approval was obtained from the Australian National University Human Research Ethics Committee (Protocol number: 2010/468).

### Data collection and analysis

Data were scanned and entered into SPSS files for analysis. On line responses were merged electronically. Analysis was undertaken using SPSS Version 19 (Somers, NY, USA) and Stata 9 (College Station, TX, USA).

The complex stratified sample structure and the variable response rates meant that separate weighting was needed for each sub-sample to allow estimates of each of the three populations, and all estimates provided here are weighted. The responding NSA sample (n = 1432) is broadly representative of older Australians, although they are somewhat more affluent and better educated than the aged population as a whole. The majority of this paper is based on the NSA sample because it is representative of older Australians [23], although the other samples are referenced where this information adds to understanding of how chronic conditions influence access.

## Results

### Sample characteristics

The number of responses completed and included was 2,540. The NSA sample included 1,432 respondents (28.6% response rate). The Lung sample included 681 respondents (22.2% response rate) and the Diabetes sample included 427 respondents (17.1% response rate).

The chronic conditions most widely reported in the NSA sample (see Table 1) were high blood pressure (41.9%), arthritis (35.0%), chronic pain (19.5%) and asthma (19.1%). The percentage of respondents who also reported ever having had a diagnosis of cancer was 25.9%, with 10.3% reporting being treated for cancer in the past three months.

**Table 1:** Proportions of people in the NSA sample (1,432 respondents) with chronic conditions who attend complementary medicine practitioners

	**Number of respondents**	**Percent of people with condition attending a complementary practitioner**		
		**Percent**	**95% confidence interval**	
Cancer	401	6.6	4.3	8.9
Recent cancer	148	8.4	3.7	13.1
Heart condition	245	8.8	5.9	11.6
High blood pressure	624	5.3	3.5	7.1
Diabetes	184	5.1	1.5	8.7
Renal condition	43	12.1	2.6	21.6
Asthma	266	6.1	2.7	9.5
COPD	60	5.9	0.4	11.4
Arthritis	520	9.2	6.7	11.8
Osteoporosis	175	15.9	10.8	20.9
Chronic pain	276	13.3	9.5	16.5
Anxiety/depression	208	12.0	6.2	17.7
Overall		8.8	7.3	10.4

### Use of CAM practitioners

Respondents were asked to identify which health professionals they had ‘received advice or treatment from in the last 3 months’, including ‘complementary health practitioners, e.g. naturopath’. Overall, 8.8% of NSA respondents reported seeing a CAM practitioner in the past three months, (12.1% of women and 3.9% of men). In comparison, 3.9% of the Diabetes sample, and 3.4% of the Lung sample, reported consulting a CAM practitioner during that period. In the NSA group, 5.6% reported between one and three visits to a CAM practitioner in the previous 3 months with 1.2% reporting 4–5 visits and 1% reporting more than 6 in the same period. Most people who consulted a CAM practitioner had also consulted a general practitioner or medical specialist within the same period, (95% of the Diabetes respondents, 100% of the Lung respondents, and 85% of NSA respondents.) Within the NSA group, 15% reported that they had seen a CAM practitioner, but not a medical practitioner, within the previous 3 months.

Table 1 presents the percentage of NSA respondents with one or more of ten chronic conditions that were included in the survey, together with the percentage of respondents with each illness who reported consulting a CAM practitioner in the past three months.

Broadly speaking the table suggests that those with pain-associated or mobility limiting conditions were more likely to consult CAM practitioners than those with other conditions. Respondents with musculoskeletal conditions such as osteoporosis, or chronic pain, were significantly more likely to have seen have seen a CAM practitioner in the previous three months than respondents with cancer (ever treated), high blood pressure, diabetes or asthma. With the relatively wide confidence intervals due to small numbers with each condition no other differences are significant, although the estimated proportions of people with depression or anxiety or with renal conditions CAM practitioner are relatively high at 12%.

Table 2 shows the percentage of each sample who reported consulting a CAM practitioner within the previous three months, by number of chronic conditions they reported.

**Table 2:** Use of CAM practitioner by number of chronic conditions and sample type

	**Number of respondents**	**Percent of people in this sample and with this number of conditions attending a complementary practitioner**		
		**Percent**	**95% confidence interval**	
**NSA Sample**				
*Number of chronic conditions*				
0	205	8.9	5.5	12.3
1	350	11.4	9.1	13.7
2	383	6.2	3.3	9.1
3	236	9.6	5.4	13.8
4	136	7.4	3.2	11.5
5 or more	122	8.6	3.3	14.0
Overall	1,432	8.8	7.3	10.4
**NDDS Sample**				
*Number of chronic conditions*				
0	7	NA		
1	49	1.3	−2.5	5.0
2	105	0.7	−1.1	2.5
3	91	4.6	−0.2	9.3
4	75	5.7	0.3	11.2
5 or more	100	7.2	2.2	12.2
Overall	427	3.9	1.9	6.0
**Lung Foundation Sample**				
*Number of chronic conditions*				
0	6	NA		
1	82	1.2	−0.7	3.1
2	129	3.1	0.2	6.0
3	125	3.2	0.1	6.3
4	134	6.0	2.6	9.5
5 or more	205	2.7	0.2	5.2
Overall	681	3.3	2.1	4.5

Although confidence intervals are very wide, there is a pattern in the Diabetes sample of increasing numbers of chronic conditions being associated with higher percentages using CAM practitioners, with a similar pattern in the Lung sample up to 4 conditions, although with smaller use of CAM practitioners in the group with most chronic conditions. However, this trend is not evident in the NSA sample, where there is no pattern that is significantly associated with number of conditions.

Table 3 shows demographic patterns of CAM practitioner users by respondents in the NSA sample.

**Table 3:** Demographic patterns of users of CAM practitioners in the NSA sample

**Category**	**Number of respondents**	**Percent of NSA members in this category attending a complementary medical practitioner**		
		**Percent**	**95% confidence interval**	
All NSA members	1,432	8.8	7.3	10.4
*Gender*				
Male	641	3.9	2.4	5.4
Female	780	12.1	9.8	14.5
*Aged*				
50-59 years	175	9.8	5.8	13.7
60-69 years	581	10.1	7.4	12.8
70-79 years	387	7.2	4.7	9.6
80 years or over	216	2.6	0.3	5.0
*Employment status*				
Employed full-time	158	6.4	2.1	10.7
Employed part-time	180	10.4	5.9	14.9
Retired	957	8.7	7.1	10.3
Home duties	71	7.7	1.0	14.3
Unemployed	8	18.4	−4.9	41.5
Other employment status	37	4.7	−1.8	11.2
*Qualifications*				
No post-school qualifications	578	6.3	4.2	8.4
Post school qualifications but no degree	491	9.6	7.5	11.7
Degree or higher degree	336	12.8	9.4	16.2
*Socio-economic status (SES) of area of residence*				
First quintile (lowest SES)	122	6.4	1.8	11.0
Second quintile	194	8.8	4.9	12.7
Third quintile	257	7.2	4.7	9.6
Fourth quintile	362	8.1	5.1	11.2
Fifth quintile (highest SES)	477	10.4	8.1	12.8
*Note some groups do not add to 1,432 due to missing values*				
*Region*				
Major Cities	57.8	9.0	7.3	10.8
Inner Regional Areas	27.7	9.3	6.2	12.4
Outer Regional Areas	11.3	7.7	4.6	10.7
Remote Areas	3.2	0.6	−4.2	5.5
*General health*				
Excellent	8.8	9.6	5.8	13.4
Very good	39.1	9.9	7.7	12.1
Good	35.3	8.5	5.9	11.1
Fair	14.0	5.2	2.3	8.1
Poor	2.8	14.7	5.2	24.3
*Number of chronic conditions*				
0	15.9	8.9	5.5	12.3
1	27.1	11.4	9.1	13.7
2	25.8	6.2	3.3	9.1
3	14.4	9.6	5.4	13.8
4	8.1	7.4	3.2	11.5
5 or more	8.5	8.6	3.3	14.0

Women were significantly more likely than men to use CAM practitioners, and the elderly (over 80 years of age) significantly less likely than those aged 50–69 years. Those with degree qualifications were more likely than those with no post school qualifications to use CAM practitioners. Respondents living in remote areas were significantly less likely than those in inner regional or metropolitan areas to report using CAM practitioners. No significant differences in use were found in relation to employment status or socio-economic status of the area of residence. There was no significant difference in CAM practitioner use in relation to self-assessed health or (as noted above) the number of chronic conditions reported.

## Discussion

This study is the only national study specifically to report use of CAM practitioners by older Australians who have chronic illness. Overall, 8.8% of our NSA sample, most closely representative of the wider population of older Australians, reported seeing a CAM practitioner in the previous three months. In common with other studies, we found that those who consult CAM practitioners are likely to be women and to have higher levels of education. However, in contrast with other studies [12,17], we did not find that living in an area of higher socioeconomic status was related to higher use. We have also been able to show a differentiation in use with a decline in use with increasing age, with those over 80 years of age being the lowest users. In contrast to the Adams study [17] where 3.1% of those over 65 years visited CAM practitioners over the past year, our NSA sample showed use by 10.1% of those aged between 60 and 69 years; and 7.2% of those aged between 70 and 79 years in the past 3 months. It was only in the group of those aged 80 and above that the rate of use fell to 2.6%.

The different methodologies used by researchers in this area make direct comparisons of rates of use difficult. Many of the studies cited report CAM practitioner use over the previous twelve months (8, 17,) rather than the 3 months used in this study and use different age groupings and include different CAM practitioners. However, the proportions of those consulting CAM practitioners in the previous 3 months, at 8.8%, is at least as great as those reported by Adams [17] and more in line with the 26.5% rate reported by MacLennan and colleagues [8].

The gender difference in use is wider in our study (almost 3 F:1 M) than in other studies that report a 2:1 difference [21] but the reasons for this are not clear.

Studies looking at specific chronic conditions have found similar CAM usage to that in our study. In our study, 8.4% of respondents with recent treatment for cancer and 8.8% of respondents with a heart condition in the NSA sample reported accessing CAM practitioners. We found higher use of CAM practitioners among people with musculoskeletal conditions such as arthritis (9.2%), osteoporosis (15.9%) and chronic pain (13.0%) than cancer and heart disease. These figures are higher than those reported by Adams et al. [17] (5.6%, 4.4% and 8.5% respectively), even when reporting a shorter period.

While Westert et al. [24] have shown that use of health services increases with increasing ill-health and numbers of co-morbid conditions, the current study found that this did not apply to the use of CAM practitioners: neither self-assessed health nor the reported increasing numbers of chronic conditions was significantly related to their reported use. This may suggest that the reasons for consulting a CAM practitioner are linked to the presence of particular conditions, so that the actual number of conditions is less important, or, as other authors have suggested that it is related to the patient ‘world view’ in making their choice of provider [16] especially when “conventional care is not relieving their symptoms” [25].

This possibility is supported by the fact that the two chronic condition samples (Diabetes and Lung), for which conventional medicine has clear guidelines and treatment models had low CAM use.

Finally, respondents living in remote areas were significantly less likely than those in inner regional or metropolitan areas to report using CAM practitioners, a finding that may be related to the limited number of CAM (and other) practitioners in Australia’s remote areas, but that contrasts with Adams’ 2003 findings [10].

### Limitations

There is no commonly used group of practitioners included under a definition of CAM practitioner, and our study left it open to respondents to include CAM practitioners according to their own views. There is no capacity in this study as a result to link particular respondent characteristics to particular practitioners. Additionally the survey did not capture whether respondents saw mainstream healthcare practitioners who have been trained in CAM [26].

Using recall as the basis for data may lead to inaccurate reporting, which we hoped to minimise by using a limited period, particularly in relation to the number of times respondents consulted different health practitioners.

Our survey did not ask respondents whether they sought care from a particular practitioner in connection with a particular chronic disease or other health issue [3]. It is possible that people with diabetes, for example, were accessing CAM practitioners for management of other conditions or to improve overall health [2].

The response rate from each of the sample groups is lower than we had hoped, which limited the analysis of all variables, and inevitably leads to a concern that these results are not representative. On the other hand, responses were received from respondents nationally, which provides a strength to the results.

### Implications for management of chronic illness

The vast majority of those who consulted CAM practitioners also consulted mainstream medical professionals, almost universally in the case of respondents from both the Diabetes group and the Lung group. Respondents with conditions that have well evidenced treatment regimes that are widely used, such as hypertension, diabetes and asthma, have the smallest percentage seeking advice or treatment from CAM practitioners. On the other hand, higher proportions of people with conditions that are complex, involve pain management, and whose effects may be intractable using conventional medicines report seeking assistance from CAM practitioners.

We suggest that these findings have particular implications for mainstream healthcare practitioners. Other studies have shown that the combination of multiple care practitioners, as well as multiple medicines affect the wellbeing of people with chronic conditions [27,28]. For this reason it is important that mainstream healthcare practitioners be aware of the healthcare choices their clients are making.

Robinson & McGrail’s literature review of disclosure of CAM and CAM practitioner usage found that the disclosure rate of usage to mainstream healthcare practitioners may be as low as 23%, with several reviewed studies reporting non-disclosure rates of 60-70% [13]. The reasons for this include, but are not limited to, patient perception that the physician does not value CAM, physician is viewed as ignorant of CAM, physician does not ask about CAM usage, patient forgets to mention CAM usage [13]. The disclosure of CAM usage to mainstream healthcare practitioners is essential, as Kiat and colleagues have suggested, to patient outcomes [1].

## Conclusion

Our data suggests in addition that since a substantial percentage of consumers with chronic conditions seek advice from CAM practitioners, it is important to consider the active role of CAM practitioners as part of overall health care planning and management. Like mainstream healthcare practitioners, CAM practitioners are likely to be in a position of trust and confidence with the consumer. The potential exists for adverse effects to result not only from competing medicines, but from competing advice. Sewitch et al. suggest that increased knowledge of CAM efficacy and increased mainstream healthcare practitioners’ knowledge of CAM therapies will “help integrate CAM into mainstream medical care” [29] at 151]. In previous SCIPPS work [30,31] we note that competing medication regimes and competing advice about care are reported as significant barriers to best care by both consumers and care practitioners. Commenting on service fragmentations, a nursing manager said:

[Consumers] often express their concern that professionals aren’t talking to each other, aren’t linking up. It’s very disheartening for them to go and see a GP in their community setting, try to see a podiatrist maybe privately, and a nutritionist somewhere else. And the question they often ask me is ‘why don’t these people ever speak to each other to co-ordinate my care?’ (31: 7).

This quote speaks of fragmentation between medical health and allied health care professionals, but such fragmentation may be even more evident with CAM practitioners, especially given the reluctance of many to declare use of CAM to their general practitioner. Improving communication and integration of health services is essential to providing comprehensive care and we suggest that the concept of the ‘treating team’ needs a broad interpretation.

We recommend that questions about the use of CAM and consultations with CAM practitioners should be raised by health care practitioners in bio-medical consultations as a matter of standard procedure. Particularly in the case of clients with osteoporosis and other conditions where pain and pain management are a feature, regular discussions about CAM therapy and practitioner use is important to foster patient safety and best multidisciplinary practice.

## Competing interests

The funding organisation (NHMRC) had no role in the study design, data collection, analysis and interpretation, or the writing and publication of this article. The authors declare that they have no competing interests.

## Authors’ contributions

LY led the development of the research questionnaire, contributed to the questionnaire design, analysis, and was the principal author of the paper. TJ contributed to the design of the research questionnaire, analysis and was a major contributor to the writing of the paper. IMcR contributed to the questionnaire design and sample selection and provided the expert statistical support for the analysis of the results. He also contributed to the writing of the paper, particularly in relation to the methods and results. All authors read and approved the final manuscript.

## Pre-publication history

The pre-publication history for this paper can be accessed here:

http://www.biomedcentral.com/1472-6882/13/73/prepub

## References

[B1] Kiat H Sun Bin Y Grant S Hsu-Tung CD Complementary medicine use in cardiovascular disease: a clinician’s viewpoint Medical J Australia 2011 195 11/12 654 656 10.5694/mja10.10976 22171853

[B2] Barraco D Valencia G Riba AL Nareddy S Draus CBSN Schwartz SM Complementary and alternative medicine (CAM) use patterns and disclosure to physicians in acute coronary syndromes patients Complement Ther Med 2005 13 1 34 40 10.1016/j.ctim.2005.02.003 15907676

[B3] Yeh GY Davis RB Phillips RS Use of Complementary Therapies in Patients With Cardiovascular Disease Am J Cardiol 2006 98 5 673 680 10.1016/j.amjcard.2006.03.051 16923460

[B4] Unantenne N Warren N Canaway R Manderson L The Strength to Cope: Spirituality and Faith in Chronic Disease J Relig Health 2011 1 15 10.1007/s10943-011-9554-9 22083464

[B5] Ernst E Prevalence of use of complementary/alternative medicine: a systematic review Bull World Health Organ 2000 78 258 266 10743298 PMC2560678

[B6] MacLennan A Wilson D Taylor A Prevalence and cost of alternative medicine in Australia Lancet 1996 347 569 573 10.1016/S0140-6736(96)91271-4 8596318

[B7] MacLennan A Wilson D Taylor A The escalating cost and prevalence of alternative medicine Prev Med 2002 35 116 173 10.1006/pmed.2002.1057 12200102

[B8] MacLennan A Myers S Taylor A The continuing use of complementary and alternative medicine in South Australia: costs and beliefs in 2004 Med J Australia 2006 184 1 27 31 16398628 10.5694/j.1326-5377.2006.tb00092.x

[B9] Sanderson CR Koczwara B Currow DC The “therapeutic footprint” of medical, complementary and alternative therapies and a doctor’s duty of care Med J Australia. [Perspective] 2006 185 7 373 376 10.5694/j.1326-5377.2006.tb00613.x 17014405

[B10] Adams J Sibritt DW Easthope G Young AF The profile of women who consult Complementary Health Practitioners in Australia Med J Australia 2003 179 297 300 12964912 10.5694/j.1326-5377.2003.tb05551.x

[B11] McLaughlin D Lui C-W Adams J Complementary and alternative medicine use among older Australian women - a qualitative analysis BMC Complement Altern Med 2012 12 1 34 10.1186/1472-6882-12-34 22471758 PMC3342907

[B12] Xue CCL Zhang AL Lin V Da Costa C Story DF Complementary and Alternative Medicine Use in Australia: A National Population-Based Survey J Altern Complement Med 2007 13 6 643 650 10.1089/acm.2006.6355 17718647

[B13] Robinson A McGrail MR Disclosure of CAM use to medical practitioners: a review of qualitative and quantitative studies Complement Ther Med 2004 12 2–3 90 98 15561518 10.1016/j.ctim.2004.09.006

[B14] Goldstein MS Brown ER Ballard-Barbash R Morgenstern H Bastani R Lee J The Use of Complementary and Alternative Medicine Among California Adults With and Without Cancer eComplementary and Alternative Medicine. [Original] 2005 2 4 557 565 10.1093/ecam/neh138 PMC1297511 16322814

[B15] Hsiao A-F Wong MD Goldstein MS Becerra LS Cheng EM Wenger NS Complementary and elaternative medicine use among Asian-American subgroups: prevalence, predictors, and lack of relationship to accultruation and access to conventional care J Altern Complementary Med 2007 12 1003 1010 10.1089/acm.2006.12.1003 17212572

[B16] Bishop FL Yardley L Lewith GT A Systematic Review of Beliefs Involved in the Use of Complementary and Alternative Medicine J Health Psychol 2007 12 6 851 867 10.1177/1359105307082447 17956965

[B17] Adams RJ Appleton SL Cole A Gill T Taylor AW Hill CL Oral complementary medicine and alternative practitioner use varies across chronic conditions and attitudes to risk Clin Epidemiol 2010 2 1 251 260 21152252 10.2147/CLEP.S12741 PMC2998815

[B18] Kristoffersen AE Norheim AJ Fønnebø VM Any difference? Use of a CAM provider among cancer patients, coronary heart disease (CHD) patients and individuals with no cancer/CHD BMC Complement Altern Med 2012 12 1 1 8 10.1186/1472-6882-12-1 22240073 PMC3305410

[B19] Greenfield S Pattison H Jolly K Use of complementary and alternative medicine and self-tests by coronary heart disease patients BMC Complement Altern Med 2008 8 47 1 8 18680571 10.1186/1472-6882-8-47 PMC2527291

[B20] Thomas KJ Nicholl JP Coleman P Use and expenditure on complementary medicine in England: a population based survey Complement Ther Med 2001 9 2 11 10.1054/ctim.2000.0407 11264963

[B21] Al-Windi A Determinants of complementary alternative medicine (CAM) use Complement Ther Med 2004 12 2–3 99 111 15561519 10.1016/j.ctim.2004.09.007

[B22] Sirois F Provider-based complementary and alternative medicine use among three chronic illness groups: associations with psychosocial factors and concurrent use of conventional health-care services Complement Ther Med 2008 16 2 74 81 10.1016/j.ctim.2007.03.006 18514908

[B23] Yen L McRae I Jeon YH Essue B Herath P The impact of chronic illness on workforce participation and the need for assistance with household tasks and personal care by older Australians Health Soc Care Community 2011 19 5 485 494 10.1111/j.1365-2524.2011.00994.x 21453439

[B24] Westert GP Satariano W Schellevis FG Van Den Bos GAM Patterns of comorbidity and the use of health services in the Dutch population Eur J Public Health 2001 11 4 365 372 10.1093/eurpub/11.4.365 11766475

[B25] Testerman JK Morton KR Mason RA Ronan AM Patient motivations for using complementary and alternative medicine Complementary Health Prac Rev 2004 9 81 92

[B26] Pirotta MV Cohen MM Kotsirilos V Farish SJ Complementary therapies: have they become accepted in general practice? Med J Australia 2000 172 105 109 10735019 10.5694/j.1326-5377.2000.tb127932.x

[B27] Braun LA Tiralongo E Wilkinson JM Poole S Spitzer O Bailey M Adverse reactions to complementary medicines: the Australian pharmacy experience Int J Pharm Pract 2010 18 4 242 244 10.1111/j.2042-7174.2010.00036.x 20636677

[B28] Elmer GW Lafferty WE Tyree PT Lind BK Potential Interactions Between Complementary/Alternative Products and Conventional Medicines in a Medicare Population Ann Pharmacother 2007 41 1617 1624 10.1345/aph.1K221 17785609 PMC2864004

[B29] Sewitch MJ Cepoiu M Rigillo N Sproule D A Literature Review of Health Care Professional Attitudes Toward Complementary and Alternative Medicine Complement Heal Pract Rev 2008 13 3 139 154

[B30] Jeon Y-H Jowsey T Yen L Glasgow NJ Essue B Kljakovic M Achieving a balanced life in the face of chronic illness Australian J Primary Health 2010 16 66 74 10.1071/PY09039 21133301

[B31] Yen L Gillespie J Jeon Y-H Kljakovic M Brien J-a Jan S Health professionals, patients and chronic illness policy: a qualitative study Heal Expect 2011 14 1 10 20 10.1111/j.1369-7625.2010.00604.x PMC5060561 20550589

